# Demand-side interventions for maternal care: evidence of more use, not better outcomes

**DOI:** 10.1186/s12884-015-0727-5

**Published:** 2015-11-13

**Authors:** Taylor E. Hurst, Katherine Semrau, Manasa Patna, Atul Gawande, Lisa R. Hirschhorn

**Affiliations:** Department of Global Health and Population, Harvard School of Public Health, 677 Huntington Avenue, Boston, MA USA; Ariadne Labs, 401 Park Drive 3 East, Boston, MA USA; Division of Global Health Equity, Brigham and Women’s Hospital, Boston, MA USA; Department of Obstetrics and Gynecology, Cambridge Health Alliance, Cambridge, MA USA; Harvard Medical School, Boston, MA USA; Department of Surgery, Brigham and Women’s Hospital, Boston, MA USA; Department of Global Health and Social Medicine, Harvard Medical School, Boston, MA USA

**Keywords:** Demand-side, Community mobilization, Financial incentives, Maternal mortality, Infant mortality, Quality improvement, Maternal care

## Abstract

**Background:**

Reducing maternal and neonatal mortality is essential to improving population health. Demand-side interventions are designed to increase uptake of critical maternal health services, but associated change in service uptake and outcomes is varied. We undertook a literature review to understand current evidence of demand-side intervention impact on improving utilization and outcomes for mothers and newborn children.

**Methods:**

We completed a rapid review of literature in PubMed. Title and abstracts of publications identified from selected search terms were reviewed to identify articles meeting inclusion criteria: demand-side intervention in low or middle-income countries (LMIC), published after September 2004 and before March 2014, study design describing and reporting on >1 priority outcome: utilization (antenatal care visits, facility-based delivery, delivery with a skilled birth attendant) or health outcome measures (maternal mortality ratio (MMR), stillbirth rate, perinatal mortality rate (PMR), neonatal mortality rate (NMR)). Bibliographies were searched to identify additional relevant papers. Articles were abstracted using a standardized data collection template with double extraction on a sample to ensure quality. Quality of included studies was assessed using McMaster University’s Quality Assessment Tool from the Effective Public Health Practice Project (EPHPP).

**Results:**

Five hundred and eighty two articles were screened with 50 selected for full review and 16 meeting extraction criteria (eight community mobilization interventions (CM), seven financial incentive interventions (FI), and one with both). We found that demand-side interventions were effective in increasing uptake of key services with five CM and all seven FI interventions reporting increased use of maternal health services. Association with health outcome measures were varied with two studies reporting reductions in MMR and four reporting reduced NMR. No studies found a reduction in stillbirth rate. Only four of the ten studies reporting on both utilization and outcomes reported improvement in both measures.

**Conclusions:**

We found strong evidence that demand-side interventions are associated with increased utilization of services with more variable evidence of their impact on reducing early neonatal and maternal mortality. Further research is needed to understand how to maximize the potential of demand-side interventions to improve maternal and neonatal health outcomes including the role of quality improvement and coordination with supply-side interventions.

## Background

Maternal mortality results in approximately 800 deaths every day [1]. In 2013, 289,000 women died from potentially preventable causes during pregnancy and childbirth, and 99 % of these deaths occurred in low and middle-income countries (LMIC) [1]. Since 1990, there has been a 45 % reduction in maternal mortality; however, limited access to quality routine and emergency care during pregnancy and delivery leaves a large number of women at risk of preventable death [2].

There are a number of factors that contribute to maternal and early neonatal mortality. The three delays model proposes that mortality can be largely attributed to a 1) delay in the decision to seek care, 2) delay in arrival at a health facility, and 3) delay in the provision of care [3]. Increasing the uptake and quality of facility-based maternal care in resource-limited settings is critical to achieving the goals of reducing maternal and early neonatal mortality. Interventions have included those focused on improving the quality and reach of services (supply) and those focused on increasing uptake (demand). Demand-side barriers hinder a woman’s choice to seek or ability to reach accessible, high quality care, including: lack of information about services/providers, perceived quality of services, direct and indirect costs, discrimination (religious, political, ethnic), household and community preferences, and decision-making autonomy [4, 5]. Demand-side interventions are designed to increase uptake through financial incentives that reduce the cost of accessing services or through community mobilization efforts to improve knowledge about available services and address cultural attitudes which may prevent uptake of potentially life-saving services [4]. This increase in uptake is a critical step in the path to reduce maternal and neonatal harm.

We conducted a review of published research and evaluations to explore the evidence supporting demand-side interventions on increasing uptake of key services and reducing maternal and neonatal mortality.

## Methods

A rapid search of PubMed was conducted using the following search terms:

“Health Services Needs and Demand”[majr] OR “Maternal Health Services/economics”[mesh] OR “Community Networks”[mesh] OR “Health Promotion”[Mesh] OR “Patient-Centered Care”[mesh] OR demand side[tiab] OR supply side[tiab] OR community mobili*[tiab] OR community engagement[tiab] OR patient cent*[tiab] OR voucher*[tiab] OR financial incentiv*[tiab] OR cash*[tiab] OR cash transfer[tiab] AND (“infant mortality”[mesh] OR infant mortality[tiab] OR neonatal mortality[tiab] OR perinatal mortality[tiab] OR peri natal mortality[tiab] OR infant death*[tiab] OR neonatal death*[tiab] OR perinatal death*[tiab] OR peri natal death*[tiab]) OR “Maternal Mortality”[Mesh] OR maternal mortalit*[tiab] OR maternal death*[tiab]

Titles were reviewed for potential relevance and abstracts of selected articles were read to identify studies for full review. To be included in the final data abstraction, the article needed to describe a demand-side intervention implemented in a low or middle-income setting, be published after September 2004 and before March 2014, describe study design (randomized controlled trial, pre-post, quasi-experimental), and report on at least one of the following outcomes: either an utilization outcome (antenatal care (ANC) visits, facility-based delivery, delivery with a skilled birth attendant) or a health outcome measure (maternal mortality ratio (MMR), stillbirth rate, perinatal mortality rate (PMR), neonatal mortality rate (NMR)).

Demand-side interventions were defined as interventions designed to increase utilization of maternal health services either through financial incentives (cash transfers, vouchers) or through the provision of information by participatory women’s groups or other community-based education efforts. Efforts to increase or improve service delivery in the community through interventions such as task-shifting or mobile clinics were considered supply-side interventions and not included. Purely qualitative studies, reviews, study protocols without results, and studies that included supply-side interventions were also excluded.

Bibliographies from relevant reviews were searched for additional studies, which were then added to the literature review. Because all identified studies were conducted in low or middle-income countries, no geographic limitations were added to the search terms. Study quality of the 16 studies included in the final review was assessed with McMaster University’s Quality Assessment Tool from the Effective Public Health Practice Project (EPHPP) [6]. Data were extracted from all eligible articles using a standard data collection form to collect information about study design, interventions, and outcome measures (Table 1). The tool was tested by dual extraction of five articles by two authors (TH and LRH) to ensure consistency. The final data extraction was done by one author (TH) with validation of identified areas of uncertainty by a second author (LRH). The completed data extraction was reviewed by two authors (LRH and MP).

**Table 1:** Description of studies included in the review

Author	Publication Year	Country	Setting	Participants	Study Design	Eligibility Criteria	Intervention	Primary Outcomes
*Community Mobilization*								
Fottrell	2013	Bangladesh	Rural	19,301 births	Cluster-RCT	Women permanently residing in the study area who had a recorded birth or pregnancy related death in the final 24 months of the intervention	Monthly peer-facilitated participatory action and learning groups where mothers discussed neonatal and maternal health problems and brainstormed ideas to address them	NMR
Hounton	2009	Burkina Faso	Rural	Intervention: 43,612 women Control: 52,126 women	Quasi-experimental	Women aged 12–49 who had been pregnant during the survey reference period	Community leaders led structured meetings with health professionals, religious leaders, and administrative officials to identify barriers to care and plan solutions	Institutional births, NMR, MMR
Lewycka	2013	Malawi	Rural	24 intervention and control clusters (Intervention: 27,361 women Control: 28,570 women)	Cluster-RCT	Women of childbearing age (10–49) that lived in the study area	Trained facilitators led community based groups to identify maternal and child health problems and solutions	MMR, PMR, NMR, IMR exclusive breastfeeding
Manandhar	2004	Nepal	Rural	Intervention clusters: 14,884 participants Control clusters: 14,047 participants	Cluster-RCT	Closed cohort of married women of reproductive age (15–49) who could become pregnant	Trained, local facilitators led women’s groups to increase knowledge and implement action for change	NMR
More	2012	Mumbai (India)	Urban (slums)	24 Intervention & control settlements 283,000 total population 18,197 births	Cluster-RCT	Women of child bearing age in intervention settlement	Series of 26 women's group sessions led by facilitator to increase knowledge develop an implement local strategies to address identified priority issues	Perinatal care, MMR, extended perinatal mortality
Mushi	2010	Tanzania	Rural	512 deliveries	Pre-post	All deliveries that occurred during the study period	Safe motherhood promoters led community groups and conduct home visits with pregnant women	Skilled birth attendance
Persson	2013	Vietnam	Rural and urban	Intervention: 44 communes Control: 46 communes	Cluster-RCT	Mother-newborn pairs in districts with NMR ≥ 15/1000	Local facilitators led monthly meetings with health workers, health center staff, and community members to prioritize perinatal health problems and solutions	NMR
Tripathy	2010	India	Rural	18 intervention and control cluster (18,775 total births)	Cluster-RCT	Women 15–49 years old who had given birth during the study period and were residing in project area	Trained, local women facilitated monthly meetings using the participatory learning and action cycle to share information, identify maternal and newborn health problems, and collectively design, implement, and evaluate strategies to address these problems	NMR, maternal depression scores
*Financial Incentives*								
Bellows	2013	Kenya	Urban (informal settlements)	4362 women	Pre-post	The 2005/06 data set included all females aged 12–54 years old who were registered in the longitudinal NUHDSS and had a live birth or stillbirth between January 2004 and December 2005. The second data set included all females aged 12–54 who had given birth in the last 6 months	Eligible women could purchase vouchers that covered antenatal care, facility-based delivery, and postnatal care	Delivery in a health facility
De Allegri	2012	Burkina Faso	Rural	1934 women	Pre-post	Women residing in the 1050 households in Nouna Health District included in the representative sample	Women who presented for a normal facility-based delivery received an 80 % subsidy, women who presented for complicated deliveries or C-sections charged proportionally higher rates	Delivery in a health facility
Gupta	2012	India	Rural and urban	Pre: 3929 women Post: 5604 women	Pre-post	All women who delivered at the NSCB Medical College & Hospital of Jabalpur district between August 2003 and August 2007. All pregnant women were eligible to receive the JSY cash incentive if they chose to deliver in a facility	Provided antenatal and postnatal services as well as a cash incentive for mothers after they delivered in a government or accredited private health facility	Maternal mortality and maternal morbidity
Ir	2010	Cambodia	Rural	2725 women	Quasi-experimental	Pregnant women who received vouchers and had a facility-based delivery in the three districts where the program was implemented	Women received vouchers for antenatal visits, facility-based deliveries, and postnatal care as well as funds for transportation costs. Health Equity Fund schemes were also in place to promote access to health services for the poor	Proportion of facility- based deliveries
Lim	2010	India	Rural and urban	182,869 women	Quasi-experimental	Women 15–44 years old included in the DLHS survey	Women received a financial incentive after delivering in a government or accredited private health facility	Perinatal death, neonatal death, MMR
Nguyen	2012	Bangladesh	Rural and urban	*16 intervention & comparison sub-districts (*1104 women in each)	Quasi-experimental	Women who had delivered 6 months prior to the survey	Women received money for transport costs and vouchers for antenatal care, safe delivery care in a facility or at home, emergency care for obstetric complications, and postnatal care. After delivery with a qualified provider women also received a cash incentive and gift box	ANC visits, institutional delivery, delivery attended by a qualified provider (facility or at home), incidence of C-section, incidence of PNC check-ups with a qualified provider
Randive	2013	India	Rural and urban	284 districts (population 1.7 million)	Pre-post	Population-based national level surveys containing maternal mortality and birth data	Women received a financial incentive after delivering in a government or accredited private health facility	MMR, institutional births
Barber	2009	Mexico	Rural	Intervention: 712 births Control: 180 births	RCT	Women eligible for *Oportunidades* (low-income household in a marginalized community) who lived in the treatment or control communities, had a singleton live birth between 1997 and 2003, and who received and reported on ONC	Households received a cash transfer if a woman attended educational programming and completed a prescribed prenatal care plan (% ANC visits and nutritional supplements)	Overall quality of care score, quality scores within three domains (history taking and diagnostics, physical examination, and prevention)

*ANC* antenatal care, *PNC* postnatal care, *RCT* randomized control trial, *MMR* maternal mortality ratio, *PMR* perinatal mortality rate, *NMR* neonatal mortality rate, *IMR* infant mortality rate

## Results

### Identified studies

**Fig. 1:**
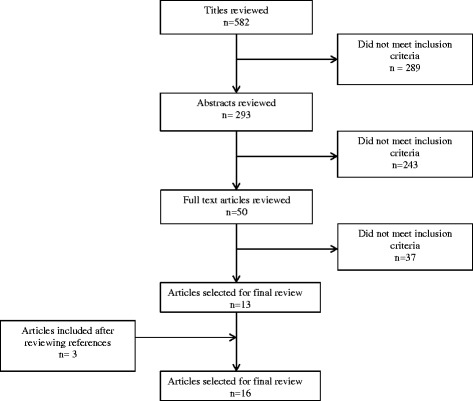
Study review for inclusion

An initial search resulted in 582 articles (Fig. 1). Following review of titles and abstracts, 50 were selected for full review with 13 studies meeting criteria for extraction. Three additional studies [7–9] were identified from reviewing the references of the articles found in the search, resulting in 16 studies in the final analysis. Of these 16 studies, eight described community mobilization interventions, seven reported on financial incentive interventions, and one intervention included both financial incentives and community mobilization. Community mobilization interventions included participatory women’s groups, training of community facilitators, and community level health promotion. Financial incentive interventions included voucher schemes and conditional cash transfers, which involve the provision of money to a household or individual if specific, pre-determined conditions are met [10]. Interventions were implemented across a range of low and middle-income countries including: Bangladesh, Burkina Faso, Malawi, Cambodia, Kenya, Tanzania, Nepal, India, Vietnam, and Mexico. Nine of the interventions were implemented in rural settings, two in urban settings, and five spanned both rural and urban settings (Table 1).

Of the 16 final articles selected, seven were randomized controlled trials, five were pre-post studies, and four were quasi-experimental study designs. When assessed using McMaster University’s Quality Assessment Tool, individual studies ranged in quality from 1 (strong) to 3 (weak). The overall average of study quality was 2.375. Outcomes measured varied between studies and included both utilization measures (antenatal care (ANC), facility-based delivery, delivery with a skilled birth attendant) and health outcome measures (maternal mortality ratio (MMR), stillbirth rate, neonatal mortality rate (NMR), perinatal mortality rate (PMR)).

### Effectiveness of interventions

#### Utilization measures

ANC visits, facility-based delivery, and delivery with a skilled birth attendant were included as service uptake measures in the extraction. Associations between demand-side interventions and utilization measures were found in a number of studies (Table 2). There were 11 studies that measured uptake of ANC, seven community mobilization interventions, three financial incentive interventions, and one with both CM and FI interventions. Eight out of the eleven (72 %) studies reported an increase in antenatal care utilization, including four of the seven community mobilization studies, all three of the financial incentive studies, and the one study reporting a combination intervention (Table 3) [9, 11–13]. For example, in rural Mexico, pregnant women who were beneficiaries of *Oportunidades* received a cash transfer conditional on attending five antenatal care visits, taking nutritional supplements, and participating in monthly meetings led by beneficiary representatives to discuss prenatal care, nutrition, and reproductive health information. Beneficiaries of the program reported receiving 12.2 % more antenatal care services than non-beneficiaries [5].

**Table 2:** Results by type of demand-side intervention

Community mobilization							
	Maternal mortality ratio	Stillbirth rate	Perinatal mortality rate	Neonatal mortality rate	Antenatal care	Facility-based delivery	Skilled birth attendance
Fottrell	NS	NS	NS	ARR: 0.62 (0.43, 0.89)	NS	NS	NS
Hounton	NS	--	OR: 0.75 (0.70, 0.80)	--	--	30 % increase	--
Lewycka	AOR: 0 · 51 (0 · 26–0 · 99)	--	NS	NS	Any ANC at health facility AOR: 1.50 (1.03 to 2.19)	NS	NS
Manandhar	AOR: 0.22 (0.05–0.90)	NS	--	AOR: 0.70 (0.53–0.94)	Any ANC AOR: 2 · 82 (1.41–5.62)	AOR: 3 · 55 (1 · 56–8 · 05)	Doctor, nurse, or midwife AOR: 3 · 53 (1 · 54–8 · 10)
More	--	NS	NS	AOR: 1.48 (1.06, 2.08)	NS	NS	--
Mushi	--	--	--	--	Early ANC booking increase from 18.7 to 56.9 %, *p* < 0.001	--	Increase in SBA from 34.1 to 51.4 %, *p* < 0.05
Persson	--	--	--	NS	AOR: 2.27 (1.07–4.80)	NS	--
Tripathy	NS	NS	AOR: 0.79 (0.69, 0.91)	AOR: 0 · 68 (0 · 59–0 · 78)	NS	NS	NS
Financial incentives							
	Maternal mortality ratio	Stillbirth rate	Perinatal mortality rate	Neonatal mortality rate	Antenatal care	Facility-based delivery	Skilled birth attendance
Bellows	--	--	--	--	Any ANC visit: OR: 16.5 (4.0–68.1)	OR: 1.4 (1.2–1.6)	OR: 1.2 (1.1-1.4)
					4 + ANC visits: OR: 1.9 (1.6–2.4)		
De Allegri	--	--	--	--	--	Increase from 49 to 84 % (*p* < 0.001)	--
Gupta	Increased (1985 to 2444 per 100,000 live births)	--	--	--	--	42.6 % increase in FBD	--
Ir	--	--	--	--	--	Increase in FBD from 16.3 to 44.9 % of the expected number of births (2.4 to 7 % for voucher recipients, 58.1 to 69.8 % for Health Equity Fund recipients)	--
Lim	NS	--	−3.7 (−5.2, −2.2)	−2.3 (−3.7, −0.9)	3 ANC visits: 10.7 % (9.1 to 12.3)	43.5 % (42.5 to 44.6) increase in FBD	36.6 % (35.6 to 37.7) increase in SBA
Nguyen	--	--	--	--	Cross sectional difference	37.5 % in intervention vs. 18.7 % in comparison areas, *p* < 0.001	63.7 % in intervention vs. 27.1 % in comparison areas, *p* < 0.001
					Any ANC check-up: 91.6 % vs. 75.6 %, *p* < 0.001		
					≥ 3 ANC check-ups 0.241		
					54.8 % vs. 33.6 %, *p* < 0.001		
					≥1 ANC visit with qualified provider 79.2 % vs. 54.9 %, *p* < 0.001		
Randive	NS	--	--	--	--	Increase from 20 to 49 %, *p* < 0.05	--
Both							
	Maternal mortality ratio	Stillbirth rate	Perinatal mortality rate	Neonatal mortality rate	Antenatal care	Facility-based delivery	Skilled birth attendance
Barber	--	--	--	--	12.2 % increase in number of recommended ANC procedures received	--	--

*NS* not significant (p-value > 0.05), *ARR* adjusted risk ratio, *OR* odds ratio, *AOR* adjusted odds ratio, *MMR* maternal mortality ratio, *PMR* perinatal mortality rate, *NMR* neonatal mortality rate, *IMR* infant mortality, *ANC* antenatal care, *FBD* facility-based delivery, *SBA* skilled birth attendance

**Table 3:** Aggregation of results of community mobilization and financial incentive interventions for utilization and health outcome measures

Utilization measures	Increase	No significant effect or decrease
Antenatal care		
* N* = 11 (7 CM, 3 FI, 1 B)	*N* = 8 (4 CM, 3 FI, 1 B)	*N* = 3 (3 CM)
Facility-based delivery		
* N* = 14 (7 CM, 7 FI)	*N* = 9 (2 CM, 7 FI)	*N* = 5 (5 CM)
Skilled birth attendant		
* N* = 8 (5 CM, 3 FI)	*N* = 5 (2 CM, 3 FI)	*N* = 3 (3 CM)
Health outcome measures	Decrease	No significant effect or increase
Maternal mortality ratio		
* N* = 8 (5 CM, 3 FI)	*N* = 2 (2 CM)	*N* = 6 (3 CM, 3 FI)
Stillbirth rate		
* N* = 4 (4 CM)	None	*N* = 4 (4 CM)
Perinatal mortality rate		
* N* = 6 (5 CM, 1 FI)	*N* = 3 (2 CM, 1 FI)	*N* = 3 (3 CM)
Neonatal mortality rate		
* N* = 7 (6 CM, 1 FI)	*N* = 4 (3 CM, 1 FI)	*N* = 3 (3 CM)

Fourteen studies measured change in facility-based delivery. These included seven community mobilization interventions and seven financial incentive interventions. Nine out of the 14 (64 %) reported an increased likelihood of a facility-based delivery associated with the intervention. These included two [12, 14] of the seven community mobilization interventions and all seven [8, 15–20] of the financial incentive interventions that measured facility-based delivery.

Eight studies measured change in deliveries conducted by a skilled birth attendant, including five community mobilization interventions and three financial incentive interventions. Five (62.5 %) out of eight total studies reported an increase in attended deliveries; two [12, 13] of the five community mobilization interventions and all three [8, 17, 18] of the financial incentive interventions.

Only four studies, one using a community mobilization intervention and three using financial incentives interventions, reported an increase across all three utilization indicators [8, 12, 17, 18]. Manandhar et al.’s study in rural Nepal used women’s groups led by trained, local facilitators to increase knowledge, and engage participants in identifying local maternal and neonatal problems and implementing strategies to address them. Their findings included an increase in ANC (AOR: 2.82 (1.41–5.62)), facility-based delivery (AOR: 3.55 (1.56–8.05)), and deliveries by a doctor, nurse, or midwife (AOR: 3.53 (1.54–8.10)) [12]. Bellows et al. found that allowing eligible women in urban Kenya to purchase vouchers that covered antenatal care, facility-based delivery, and postnatal care also resulted in an increase in all three measures: ANC (OR: 16.5 (4.0–68.1)), facility-based delivery (OR: 1.4 (1.2–1.6)) and skilled birth attendance (OR: 1.2 (1.1–1.4)) [8]. Lim et al. described an increased probability of attending three antenatal care visits (10.7 %), having a facility-based delivery (43.5 %), and giving birth with a skilled attendant (36.6 %) when a post-delivery financial incentive was provided to women in rural India as part of the national Janani Suraksha Yojana conditional cash transfer scheme [17]. In the fourth study conducted by Nguyen et al., Bangladeshi women received money for transport costs, vouchers for antenatal care, safe delivery at home or in a facility, emergency care for complications, postnatal care, plus a gift and cash incentive for delivery with a qualified provider. The women who received the intervention had a 13.6 percentage point increase in institutional delivery and 46.4 percentage point increase in use of a skilled provider during delivery when compared to women in the comparison sub-districts [18].

#### Health outcome measures

The association between demand-side interventions and health outcome measures was more varied. Eight studies measured MMR, five community mobilization and three financial incentive interventions. Only two studies (25 %), both community mobilization interventions, reported a drop in MMR [11, 12, 14]. Lewycka et al. found a drop in MMR (AOR: 0.51 (0.26–0.99)) associated with women’s groups in Malawi when adjusting for parity, socioeconomic quintile, baseline measures [8] In contrast, Gupta et al. reported mixed results on maternal mortality in their study on the impact of Janani Suraksha Yojana (JSY) in India. The overall maternal mortality ratio increased from 1985 to 2444 per 100,000 live births in the study population. However, when MMR was analysed by subgroup, there was a significant decrease in maternal mortality among urban women (2456 to 1710 per 100,000 live births, *p* < 0.01), an effect not seen in women from rural areas [14].

Four studies measured stillbirth rate, with none reporting a significant change after the implementation of a demand-side intervention. Six studies measured PMR, one using financial incentives and five using community mobilization interventions. Three of the six studies (50 %) reported a significant decrease in PMR, which included one [17] of the three studies using financial incentives and two [14, 21] of the three studies using community-focused efforts. Neonatal mortality ratio (NMR) was measured in seven studies, six on community mobilization interventions and one on financial incentives. Four (57 %) reported a decrease in NMR, including three community mobilization and one financial incentive intervention [12, 21, 22]. For example, Fottrell et al. reported a 38 % reduction in the neonatal mortality rate (21.3 neonatal deaths per 1000 live births versus 30.1 per 1000 in control areas) after increasing coverage of community-based participatory action and learning groups [18]. The evaluation of India’s Janani Suraksha Yojana conditional cash transfer program also found a reduction of 2.3 (0.9–3.7) neonatal deaths per 1000 live births associated with JSY payment [17]. In contrast, More et al. reported an increase in NMR (AOR: 1.48 (1.06, 2.08)) in their study of participatory women’s groups in Mumbai [23].

#### Combined utilization and health outcomes

Ten studies reported on utilization and health outcome measures, seven community and three financial interventions. Four (40 %) of these studies reported both increased utilization and decreased mortality, including three [9, 11, 13] of the seven community mobilization interventions and one [17] of the three financial incentive interventions. In rural Burkina Faso, Hounton et al. found a 30 % increase in facility-based delivery and decrease in PMR (OR: 0.75 (0.70–0.80)) during a study of community-led meetings with key stakeholders including traditional leaders, health professionals, and religious leaders [14]. In rural Malawi, facilitator-led community-based groups designed to identify maternal and child health problems and solutions were associated with both an increase in antenatal care (AOR: 1.50 (1.03–2.19)) and decrease in MMR (AOR: 0.51 (0.26–0.99)) [11]. Similarly, Manandhar et al. found that a participatory women’s group in rural Nepal was associated with an increase in antenatal care, facility-based delivery, and skilled birth attendance as well as a decrease in the maternal mortality ratio and perinatal mortality rate [12]. An evaluation of JSY in India by Lim et al. also found improvements in the use of antenatal care, facility-based delivery, and skilled birth attendance and a decrease in perinatal and neonatal mortality at the district level [17].

This association between interventions and measured utilization and health outcomes was not always observed. Two community mobilization studies [21, 22] reported improved health outcomes without any significant changes in utilization and two studies (one each of financial incentives [19] and community mobilization [9]) reported increased utilization with no significant changes to maternal or infant mortality. One of the studies of JSY found an increase in facility-based deliveries simultaneous with an increased MMR in rural areas, and decreased MMR in urban areas [15].

## Discussion

Our review of the literature published in the last decade found evidence that financial incentives and community mobilization interventions can be effective in increasing the uptake of key maternal health services, including ANC visits, facility-based delivery and delivery with a skilled birth attendant. This increase in utilization of facility-based services is an important first step in reducing maternal and neonatal morbidity and mortality. These findings are largely consistent with existing reviews that have found an increase in health service utilization after the implementation of conditional cash transfer [24] and voucher programs [25].

The overall effect of demand-side interventions on health outcome measures was less clear than the results with increasing utilization of services. While there were four studies that reported both an increase in utilization and decrease in at least one mortality outcome, [11, 12, 14, 17], another study on participatory women’s groups showed a decrease in maternal and neonatal mortality despite no increase in utilization [26]. The authors of this study suggest that this seemingly contradictory finding reflects the complex mechanisms through which community mobilization may improve maternal and child health.

Similar heterogeneity of results was seen in a study on the large-scale financial incentive scheme in India which reported an increase in facility-based deliveries and drop in neonatal mortality, but no similar drop in maternal mortality [17]. The authors hypothesized that the decrease in NMR may have resulted from the encouragement of timely care seeking that shifted mortality from stillbirth to early neonatal death. These variable effects on health outcomes are consistent with a systematic review by Prost et al., which found a significant reduction in neonatal mortality but non-significant reductions in stillbirths and maternal mortality associated with participatory learning and action groups [27].

Only one quarter of the studies that measured MMR found a decrease, with reducing neonatal mortality an even greater challenge. The weak association between increased uptake of maternal health services and health outcome measures may be explained by the quality and effectiveness of care received in health facilities [28]. Poor quality care will not translate to better health outcomes even if there is increased utilization of services. For example, a study of India’s Janani Suraksha Yojana program reported a 42.6 % increase in facility-based delivery with a statistically significant increase in overall and rural MMR, but a significant decrease in urban MMR [15]. Further exploration is needed about whether worse quality care or weaker referral systems in rural versus urban facilities may account for the difference in mortality rates. Through a series of interviews with policy representatives of the National Rural Health Mission in India, Bhattacharyya et al. noted the challenges faced in providing quality facility-based care due to limited human resources, gaps in supplies and infrastructure and poor monitoring. Although the JSY program generated demand for services, limitations in infrastructure, human resources, supplies and equipment resulted in congestion and, in some cases, a deterioration of quality of care [29].

The variability in impact observed across studies highlights the need to understand and address the complete pathway from utilization to improved health outcomes including the potential benefits of combining demand-side with supply-side interventions targeting care once a woman reaches a facility. Poor quality of facility-based care has been identified as one of the factors contributing to maternal mortality and morbidity, highlighting the need to simultaneously increase utilization and invest in developing health systems that can offer quality care to meet the increased demand for services [30]. Other factors affecting maternal and neonatal health outcomes may include delays in care seeking, or underlying causes such as lack of education, poor water and sanitation, and malnutrition [31].

The intensity and coverage of demand-side interventions within a population may also affect whether changes in health outcomes are observed. Azad et al. found no statistically significant effects on health outcome measures or utilization of services with a community mobilization program in rural Bangladesh [32]. However, when this same intervention was increased in intensity from one women’s group per 1414 population to a coverage of 1 women’s group per 309 population, a 38 % reduction in NMR in the intervention areas was seen compared to the control areas (ARR: 0.62 (0.43, 0.89)) [22]. This coverage threshold is consistent with the findings that a participatory learning and action model is a cost-effective strategy to improve maternal and neonatal survival when at least a third of pregnant women participate in the intervention [27].

Although increasing coverage of maternal health programs is important, focusing on “effective coverage” by listening to patients’ reported quality of care received, ensuring adequacy of trained staff, infrastructure, supplies and equipment, and investing in patient records needs to be prioritized to ensure that the care women and their infants receive is effective and evidence-based [33, 34]. In a study on maternal health care in Ghana, researchers found that despite increased coverage of free services, women who had negative experiences while seeking care such as overcrowding, delays, and substandard care were unlikely to seek further maternal health services [35]. Furthermore, an observational study by Chaturvedi et al., reported poor quality care in Indian health facilities under the JSY scheme. These findings underscore the importance of ensuring the availability of quality obstetric care prior to increasing demand for services. Perceptions of poor quality and experiences of care, including disrespectful treatment, have also been found to influence women’s decision of whether to seek care. For example, in a study in rural Tanzania by Kruk et al., 40 % of women who delivered in a facility bypassed their nearest facility. One of the reasons cited for this choice was perception of poor quality care [36].

More specifically, there needs to be a focus on improving comprehensive emergency care in order to improve maternal and child health outcomes [37]. Indicators for emergency obstetric care include measures of availability, geographical distribution of facilities, proportion of births in emergency facilities, ability to treat women with complications, caesarean sections and direct obstetric case fatality rate. Key functions such as administering parenteral antibiotics and performing basic neonatal resuscitation are included in the basic services that should performed, signalling the importance of investing in supply side interventions [38]. We do not have information for all studies regarding the specific services provided, or the quality of those services. A lack of technical quality of services available in health facilities may explain the gap between increases in demand and improved health outcomes. It is important for future studies on supply or demand-side interventions to collect information about the quality measures of services provided.

There are a number of limitations of this review. The settings of the studies varied widely in their national and local contexts. These include: the degree of urbanization, population and facility level targeted, existing health infrastructure, differences in geographical accessibility to services, community perceptions of the value of maternal health services and quality of existing facility-based care. These contextual factors are critical to the success of interventions and may limit the generalizability of some of the successful initiatives and explain the challenges of others. Because of the limited number of studies and the variability of both intervention design and context, we could not explore if the differences in measured change in health care uptake and outcome measures across interventions were due to the differences in intervention type, contextual factors or a combination of both.

Due to heterogeneity in intervention, study design, location, and outcomes measured, we were not able to combine results within the two categories to estimate overall intervention effect or draw conclusions on the relative effectiveness of community mobilization versus financial incentives. The majority of studies were set in India and Southeast Asian countries, so the ability to generalize across other key regions including Africa (three studies) and central and Latin America (one study) is also limited. More broadly, the use of health outcome measures such as maternal mortality ratio to detect impact may be limited by factors such as small numbers of maternal deaths, insufficient sample size, and quality of the mortality data available. Varying definitions of the outcome of interest may also impact the comparability of the results. Another limitation is the small proportion of studies that measure maternal and neonatal morbidity health outcomes. For every death that occurs, there are many more women who face long-term sequelae of pregnancy and childbirth. Understanding the impact of demand and supply side interventions on morbidity would give us a clearer picture of health outcomes.

It was also not possible to identify if the causes for variability in measured change in health outcome measures were due to the differences in intervention, contextual factors not reported (such as quality of care or presence of other demand or supply-side interventions active during the study period) or a combination of both. For example, there were varying models of community mobilization interventions. While they all aimed to facilitate a participatory learning model, there may have been variation in the facilitator or structure of community mobilization interventions, which may influence women’s decision whether to seek services. Conclusions about the impact of financial incentives were also limited by the small number of published studies, with three of the seven from India evaluating the JSY national program.

Within interventions, our review was limited to those studies which were specifically targeted at increasing demand for facility-based maternal health services (antenatal and delivery care). Other approaches to reducing women’s financial barriers to care in the context of broader financial reforms, such as health insurance schemes and financial interventions targeting non-facility-based care, are not explored in this study. As a result we may have missed the impact of broader insurance schemes as an effective strategy for increasing demand for maternal care. Although the articles that were included in this rapid review focused primarily on financial or knowledge barriers, we recognize that there may be other interventions in place to address the numerous barriers to maternal health services that did not meet our inclusion criteria or that may not be described in the peer-reviewed literature.

We only included studies that had undergone peer-review as a measure of methodological rigor, and, as a result, excluded reports of other demand-side interventions only available in the grey literature. During study selection we conducted an assessment of the quality of each of the studies using McMaster University’s Quality Assessment Tool from the Effective Public Health Practice Project (EPHPP). There was a range of reporting rigor and study designs, resulting in variation of study quality (from ‘weak’ to ‘strong’) that may limit the conclusions able to be drawn. Our methods were also designed to only include quantitative studies in order to facilitate a rapid review. The exclusion of qualitative studies limits the understanding of mechanisms through which interventions did or did not change health care utilization or outcomes. Integration of qualitative studies into the rapid reviews process can highlight potential areas for strengthening the adaption and implementation of effective interventions in different contexts and improving tested ones, which fell short of their planned goals.

Due to resource constraints, two independent reviewers only did a sample of the abstractions, with one main reviewer completing the work. However, two of the investigators reviewed the final abstractions and provided input regularly throughout the study for any areas where consultation was needed.

We limited the review to only publications from the last 10 years, and while we may have missed some published studies, we chose to limit this to reflect a rapid review approach and focus on interventions which were from the same time period as the decline in maternal mortality seen in the last decade. Another limitation is the exclusion of relevant articles that have been published since the review was conducted in March 2014, which may have added evidence for or against the effectiveness of exclusively demand-side interventions on utilization and outcomes.

## Conclusions

In conclusion, we found evidence that demand-side interventions using financial incentives or community mobilization can increase utilization of facility-based services for pregnant women with more varying evidence of impact on reducing early neonatal and maternal mortality. Further research is needed to understand the associated costs, potential for sustainability, and relative prioritization of these demand-side interventions compared with other approaches to increase uptake of these essential services for women and their newborn children. In addition, more work is needed to understand the contextual factors associated with the variable impact on maternal and neonatal mortality and the potential role of simultaneous investment in supply side factors, [4] such as staff, medical equipment and supplies, referral systems and quality of care delivered. Only by identifying and implementing locally adapted effective approaches which address gaps of demand and supply can we ensure that the growing number of women seeking facility-based care receive high quality maternal health services needed to reduce maternal and neonatal morbidity and mortality [26].
